# Optimization of a Coastal Environmental Monitoring Network Based on the Kriging Method: A Case Study of Quanzhou Bay, China

**DOI:** 10.1155/2016/7137310

**Published:** 2016-09-29

**Authors:** Kai Chen, Minjie Ni, Minggang Cai, Jun Wang, Dongren Huang, Huorong Chen, Xiao Wang, Mengyang Liu

**Affiliations:** ^1^Coastal and Ocean Management Institute, Xiamen University, Xiamen 361102, China; ^2^Fujian Provincial Key Laboratory for Coastal Ecology and Environmental Studies, Xiamen University, Xiamen 361102, China; ^3^State Key Laboratory of Marine Environmental Science, Xiamen University, Xiamen 361102, China; ^4^College of Ocean and Earth Science, Xiamen University, Xiamen 361102, China; ^5^Monitoring Center of Marine Environment and Fishery Resource, Fujian Province, Fuzhou 350003, China

## Abstract

Environmental monitoring is fundamental in assessing environmental quality and to fulfill protection and management measures with permit conditions. However, coastal environmental monitoring work faces many problems and challenges, including the fact that monitoring information cannot be linked up with evaluation, monitoring data cannot well reflect the current coastal environmental condition, and monitoring activities are limited by cost constraints. For these reasons, protection and management measures cannot be developed and implemented well by policy makers who intend to solve this issue. In this paper, Quanzhou Bay in southeastern China was selected as a case study; and the Kriging method and a geographic information system were employed to evaluate and optimize the existing monitoring network in a semienclosed bay. This study used coastal environmental monitoring data from 15 sites (including COD, DIN, and PO_4_-P) to adequately analyze the water quality from 2009 to 2012 by applying the Trophic State Index. The monitoring network in Quanzhou Bay was evaluated and optimized, with the number of sites increased from 15 to 24, and the monitoring precision improved by 32.9%. The results demonstrated that the proposed advanced monitoring network optimization was appropriate for environmental monitoring in Quanzhou Bay. It might provide technical support for coastal management and pollutant reduction in similar areas.

## 1. Introduction

Coastal areas provide important habitats for different species of organisms, including human beings [1]. Due to intensive human activities (e.g., sewage discharge, excessive fertilizer use, aquaculture, and oil spills), coastal areas face serious ecosystem pressure, which is reflected by lower pH and dissolved oxygen, and high levels of nutrients and petroleum hydrocarbons in the seawater [2–4]. To achieve sustainable development, it is necessary to formulate measures to control pollution sources and protect the coastal environment [5, 6]. Coastal management depends on reliable information about changes in the coastal environment and the causes of those changes [7, 8]. To guide the best practices for coastal management, therefore, environmental monitoring networks were set up to evaluate the conditions of the coastal environments and to obtain reliable environmental information [9–12].

In practice, high quality monitoring information plays the most important role in making possible effective and confident decisions by decision-makers and justified interpretations by scientists [10, 13–15]. Because of its fundamental role, coastal monitoring is listed on scientists' priorities of global coastal research questions [16]. However, many of the existing coastal environmental monitoring networks are ineffective and often criticized as being unscientific, too expensive, and wasteful [7, 17]. To assure its effectiveness, a coastal environmental monitoring network should be well designed or optimized, to enable the reliability of the monitoring information and to improve the efficiency of the existing monitoring network with a limited number of sites [9]. Moreover, optimal design of the monitoring network can contribute to meeting monitoring precision requirements within budgetary constraints. Coastal sampling and parameters measuring are expensive and this should be taken into account in monitoring network optimization.

At present, only a few design approaches concerning monitoring network optimization are reported, such as multivariate statistical analysis, time series analysis, information entropy, Kalman filtering, and the Kriging method [18–22]. These approaches are not equally suitable and reliable for a particular region, especially coastal area. Multivariate statistical analysis (e.g., principal component analysis, cluster analysis, and discriminant analysis) is simple and reasonable to eliminate the redundant monitoring sites, but this method may lead to the loss of key information in coastal monitoring [19, 23]. Time series analysis gives undesirable results that only optimize the monitoring frequency and cannot improve the setting of monitoring sites [24]. The defects of information entropy and Kalman filtering come from the large data requirement and complex calculation process, which means that it is usually difficult to apply them in coastal monitoring network optimization [20, 23]. Compared with these methods, the Kriging method is easy to operate, has a low cost, and is highly reliable for monitoring optimization of the coastal environment, where ecological variables are usually spatially autocorrelated [25–27].

The Kriging method is a group of geostatistical techniques to predict the value of a field at an unobserved location from observations of its value at nearby locations [27, 28]. The theoretical basis of the method was originally put forward by Krige [29], and the method was developed and empirically studied by Matheron [30]. In 1981, this method was further developed and first applied for designing monitoring networks by Hughes and Lettenmaier [31]. The result of the Kriging variance analysis is related rather to the amount and space layout of monitoring sites than the observed values. Therefore, the variance is smaller, the monitoring precision is better, and the monitoring information is more reliable. Since the 1980s, the Kriging-based monitoring network optimization approach is widely used in various aquatic environments, such as lakes, streams, rivers, groundwater, estuaries, and marine waters [23, 28, 32–36].

Quanzhou Bay is located in the coast of southeastern China and in the middle of the western side of the Taiwan Strait. The Quanzhou Bay surrounding area is one of the most actively and rapidly developing regions in China and, unfortunately, the coastal zone around Quanzhou Bay is seriously threatened by this rapid urbanization and industrialization. This area is the receptacle of several and various pollution sources, with intensive human activities having significant impacts on the marine environment [37, 38]. Owing to the outdated monitoring network and serious ecological pressure, an optimized monitoring network is necessary to meet the current coastal management needs. The aim of this study was to propose an appropriate optimized design for an environmental monitoring network for Quanzhou Bay. The ordinary Kriging method with the specific semivariogram model together with the results of the Trophic State Index was used to evaluate and optimize the existing environmental monitoring network of Quanzhou Bay. We hope that the optimized monitoring network will help to improve management efficiency for pollutant control and the environmental protection of Quanzhou Bay.

## 2. Research Area and Methods

### 2.1. Research Area

Quanzhou Bay is a semienclosed bay (24°45′–24°55′N, 118°35′–118°55′E) located in the southeast coast of China, with its mouth opening towards the Taiwan Straits. The total area of Quanzhou Bay is about 136.4 km^2^ including the intertidal area of 89.8 km^2^, and most of the bay's depth is less than 10 m [42]. The ecosystem of intertidal zones has been destroyed because of the rapid development of industrialization, agriculture, mining, and aquaculture activities in the regions surrounding Quanzhou Bay during the past few decades. Recently, the government of Quanzhou City has decided to restore the ecosystem of Quanzhou Bay and its adjacent areas. There are two rivers, the Jinjiang and Luoyang Rivers, entering Quanzhou Bay, with the former's flow being far more than the latter's.

**Figure 1:**
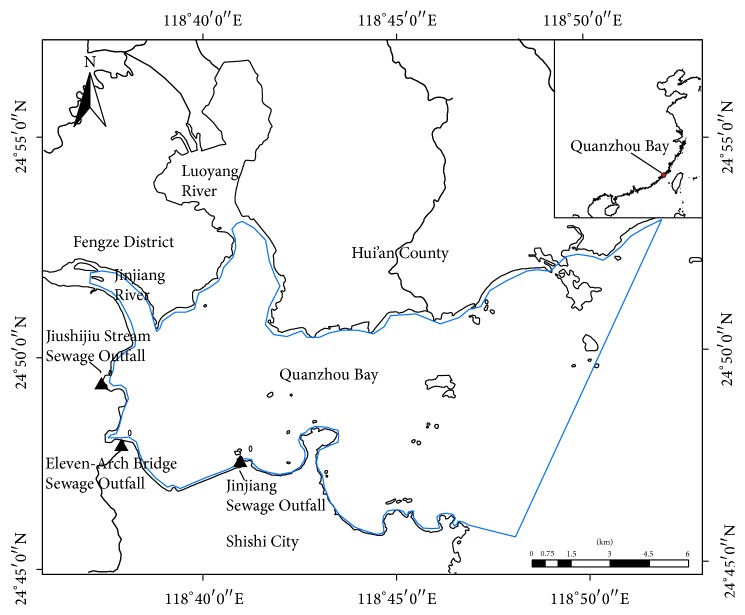
The geographical location of Quanzhou Bay.

Nearly 8 million people live around Quanzhou Bay, where the most developed coastal industrial areas contribute the largest portion of GDP in Fujian Province. With population growth and rapid economic expansion, a large number of domestic and industrial wastewater discharge points led to bay ecosystem deterioration and coastal habitat loss [42–44]. The main pollutants are mostly land-based ones, and there are three main outfalls along with the south side of the bay, the Jinjiang, Eleven-Arch Bridge, and Jiushijiu Stream Sewage Outfall, as shown in Figure 1. As the main receiving waters containing pollutants from the surrounding area, the total loads of NH_3_-N, TP, and COD discharged into Quanzhou Bay are estimated to be approximately 888.3, 130.6, and 14527.4 t/a, respectively, in 2008 and 1518.6, 558.8, and 19986.7 t/a in 2012 [38]. Owing to the limited bay self-purification ability, excessive land-based pollutants entering the bay cause ecological damage, such as the water quality deterioration, fishery resources recession, coastal habitat destruction and loss, and frequent red tide occurrence [15, 45, 46].

### 2.2. Research Methods

#### 2.2.1. Data Collection

With the rapid development around Quanzhou Bay, industrial wastewater, domestic sewage, livestock and poultry farming pollutants, and agricultural chemical fertilizer have increased greatly. This has resulted in a corresponding increase in the amount of pollutants entering the bay. Our analysis of the environmental status of Quanzhou Bay is based on 2009–2012 monitoring data from the Fujian Marine Environment and Fishery Resource Monitoring Center. The seawater quality parameters chosen were chemical oxygen demand (COD), dissolved inorganic nitrogen (DIN, including nitrate, nitrite, and ammonia), and active phosphate (PO_4_-P). COD was measured using the alkaline potassium permanganate method, nitrate was measured using the Zn-Cd reduction method, nitrite was measured using the hydrochloride naphthalene ethylenediamine spectrophotometric method, ammonia was measured using the hypobromite oxidation method, and PO_4_-P was measured using the phosphorus molybdenum blue spectrophotometric method. The frequency of monitoring was four times per year (January, April, August, and November) to estimate the water quality during the four seasons. The average of each water quality parameter at each site was used to guide monitoring network optimization.

#### 2.2.2. Trophic State Index

The spatial variability of key ecological indicators can be used to guide the appropriate coastal monitoring network design [47] and, because of the high level of nutrients in Quanzhou Bay and the requirement for land-based pollution control [38], the Trophic State Index is selected as the key ecological indicator. The Trophic State Index (*E*) is a multiparameter method proposed by Jingzhong et al. [48], which has been widely applied to evaluate the coastal water quality, calculated using the following formula:

(1) $$\begin{array}{c} E=\frac{C_{COD}\times C_{DIN}\times C_{\text{P}{\text{O}}_{\text{4}}\text{-P}}}{a}, \end{array}$$

where *C*_COD_, *C*_DIN_, and *C*_PO_4_-P_ are measured concentrations (as mg/L) of COD, DIN, and PO_4_-P, respectively. Generally, the constant *a* in the formula is the product of threshold concentrations of COD, DIN, and PO_4_-P in a specific sea area which for calculation here is 4.5 × 10^−3^. Considering that the threshold concentrations of COD, DIN, and PO_4_-P differ in the study areas, *C*_COD_′ × *C*_DIN_′ × *C*_PO_4_-P_′ is put forward instead of the constant of *a*:

(2) $$\begin{array}{c} E=\frac{C_{COD}\times C_{DIN}\times C_{\text{P}{\text{O}}_{\text{4}}\text{-P}}}{C_{{COD}_{}}^{\prime }\times C_{DIN}^{\prime }\times C_{\text{P}{\text{O}}_{\text{4}}\text{-P}}^{\prime }}, \end{array}$$

where *C*_COD_′, *C*_DIN_′, and *C*_PO_4_-P_′ are the threshold concentrations of COD, DIN, and PO_4_-P. In most research, the critical value for *C*_COD_′ is 1–3 mg/L, *C*_DIN_′ is 0.2–0.3 mg/L, and *C*_PO_4_-P_′ is 0.01–0.03 mg/L. Because of the rich nitrogen but phosphorus deficiency in the coastal waters of Fujian Province, China, we determined *C*_COD_′ as 3 mg/L, *C*_DIN_′ as 0.3 mg/L, and *C*_PO_4_-P_′ as 0.03 mg/L in Quanzhou Bay based on the related research results [49]. When the *E* value is greater than or equal to 1, the seawater is considered to suffer from eutrophication.

#### 2.2.3. Kriging Interpolation Method

Covariance and variance function are the two basic functions that are established in terms of the theory of regionalized variables. As one of the main geostatistics methods, the Kriging method is an interpolation method based on variance function theory and structural analysis. Kriging interpolation is through the sum of adjacent known sample points weighted to obtain the interpolation point value. Statistically, the method shows that the values of regional variables are unbiased. Optimal estimation in a limited area starts from the variable correlation and variability. The spatial distribution of the data for optimal linear nonbias is estimated. The Kriging method is suitable for regional variables which have a spatial autocorrelation [25, 27].

Assuming that *x* is the study of any point within the region, *Z*(*x*) is the point observed value, and there are *n* observed points in the investigated area, named *x*_1_, *x*_2_,…, *x*_*n*_. For the arbitrary unobserved points or blocks, the estimated value of *Z*_*v*_^*∗*^(*x*) is represented by the linear combination of *n* effective observed values *Z*_*v*_^*∗*^(*x*_*i*_)  (*i* = 1,2,…, *n*) within its influence scope [50]:

(3) $$\begin{array}{c} Z_{v}^{*}\left(\;x_{0}\;\right)=\overset{n}{\underset{i=1}{\sum }}\lambda_{i}Z\left(\;x_{i}\;\right), \end{array}$$

where *Z*_*v*_^*∗*^(*x*_0_) is the estimated value at the point *x*_0_; *λ*_*i*_ and *Z*(*x*_*i*_) represent the weight and observed values at point *x*_*i*_; and *Z*(*x*_*i*_) represents the Trophic State Index in this study, and the weight is endowed with the values of the surrounding observed points. The variables should be linear, unbiased, and optimally estimated [51]. When calculating the weight coefficient, this function must meet two conditions: (1) unbiased estimation of *Z*_*v*_^*∗*^(*x*), namely, the deviation of mathematical expectation, is zero; and (2) optimal estimation of *Z*_*v*_^*∗*^(*x*), namely, the variance between the estimated value of *Z*_*v*_^*∗*^(*x*) and the actual value of *Z*_*v*_(*x*), should be minimum.

The Kriging variance *σ* ^2^ can be calculated as follows [50]:

(4) $$\begin{array}{c} \sigma^{2}=\overset{n}{\underset{i=1}{\sum }}\lambda_{i}\gamma \left(\;x_{i},x_{0_{}}\;\right)+\mu , \end{array}$$

where *σ* ^2^ is the Kriging variance; *γ*(*x*_*i*_, *x*_0_) is the semivariogram between *x*_*i*_ and *x*_0_; and *μ* is the Lagrange multiplier. When *σ* ^2^ becomes smaller, the spatial distribution of the monitoring sites is more reasonable, and the monitoring network can obtain much more information of the study area. The calculation of the Kriging variance could be chosen to optimize the coastal environmental monitoring network.

We applied Geographical Information System software with the Kriging method, to calculate the average standard deviation of the estimated error of the Trophic State Index in Quanzhou Bay and to further evaluate and optimize the precision of the monitoring network in the study area. When the variation rate of average standard deviation reaches the maximum value and meets a relatively high monitoring precision in a certain range, the monitoring network can be considered as cost-effective.

## 3. Results and Discussion

### 3.1. Assessment of the Trophic State in Quanzhou Bay

Tables 1 and 2 show that the concentrations of DIN and PO_4_-P in the seawater of Quanzhou Bay were not very good from 2009 to 2012. The DIN concentrations in all observed sites could not meet the state standard of Grade IV seawater quality. Most of the observed values of PO_4_-P belonged to Grades III and IV seawater quality, and those in the Jinjiang River estuary and sewage outfall were worse than Grade IV. Compared with DIN and PO_4_-P, the observed values of COD in all sites were acceptable as a result of the bay's high environmental capacity [38].

**Figure 2:**
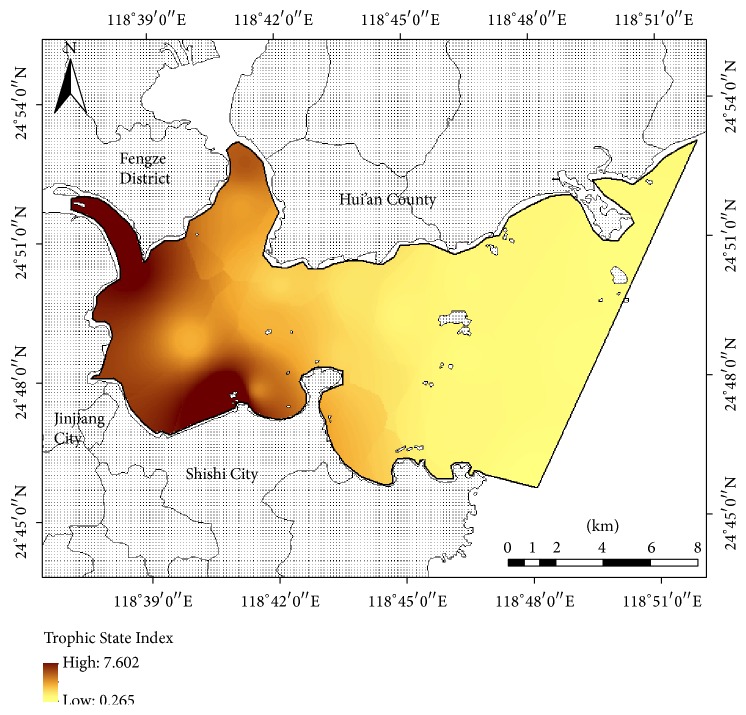
Inverse distance weighted map of the Trophic State Index in Quanzhou Bay.

The results of the Trophic State Index in Quanzhou Bay are shown in Figure 2. The *E* values in Sites 1–8 were greater than 1 (ranging from 1.124 to 7.602) and can be considered as having eutrophication status. This resulted from the developed industry zone and the large population living around the bay and the amount of nutrients from runoff transport and multiple outfall drainage discharged into the bay. The high *E* values, which appeared in the estuary and the south side of the inner bay, coordinated with the nutrient sources and the poor capacity of seawater exchange [52–54]. Zhao et al. [38] find that about 70% of the COD loading and about 85% of the DIN loading and PO_4_-P loading are from domestic and rural sewage. In addition, the nutrients from benthic release are an important source based on the weak hydrodynamic conditions [55]. Sites 9–15 in the outer bay, with the lower *E* values ranging from 0.265 to 0.621, could be considered as having oligotrophic status, as a result of the better hydrodynamic conditions accelerating water self-purification. Overall, the *E* value of Quanzhou Bay became lower from the inner to the outer bay.

### 3.2. Preliminary Optimization of the Monitoring Network of Quanzhou Bay

#### 3.2.1. Determination of the Semivariogram Model

In spatial statistics, the semivariogram is a function used to quantitatively describe the degree of spatial random field or the stochastic process. The empirical semivariogram is used as an estimator of the semivariogram needed for spatial interpolation in the Kriging method. Models of the empirical semivariogram include mainly circular, spherical, tetraspherical, pentaspherical, exponential, and Gaussian models [28]. For the Kriging method, the criteria of prediction error, including Mean Standardized, Root-Mean-Square, Average Standard Error, and Root-Mean-Square Standardized, are usually used to determine the optimal semivariogram model. If the Mean Standardized is closest to 0, the Root-Mean-Square is the minimum, the Average Standard Error is closest to the Root-Mean-Square, and Root-Mean-Square Standardized is closest to 1, the semivariogram model is optimal. The selected model influences the estimation of the monitoring sites, particularly when the shape of the curve is significantly different from the origin. The steeper the curve near the origin is, the more influence it will have on the estimation of the neighboring units [56]. In our study, the four most common semivariogram models were chosen to determine the most optimal semivariogram mode: circular, spherical, tetraspherical, and exponential.

Based on the “fitting” results of the semivariogram modeling shown in Table 3 and the judgement criteria mentioned previously, the spherical model had the best fitting performance (all criteria were optimal except the criterion of Average Standard Error). This meant that the spherical model had a relatively superior capacity to capture the characteristics of the spatial structure of the coastal environmental monitoring network. Therefore, we used the Kriging method based on the spherical model as the semivariogram for the coastal environment monitoring network optimization in Quanzhou Bay.

#### 3.2.2. Preliminary Results from the Optimization of the Monitoring Network

**Figure 3:**
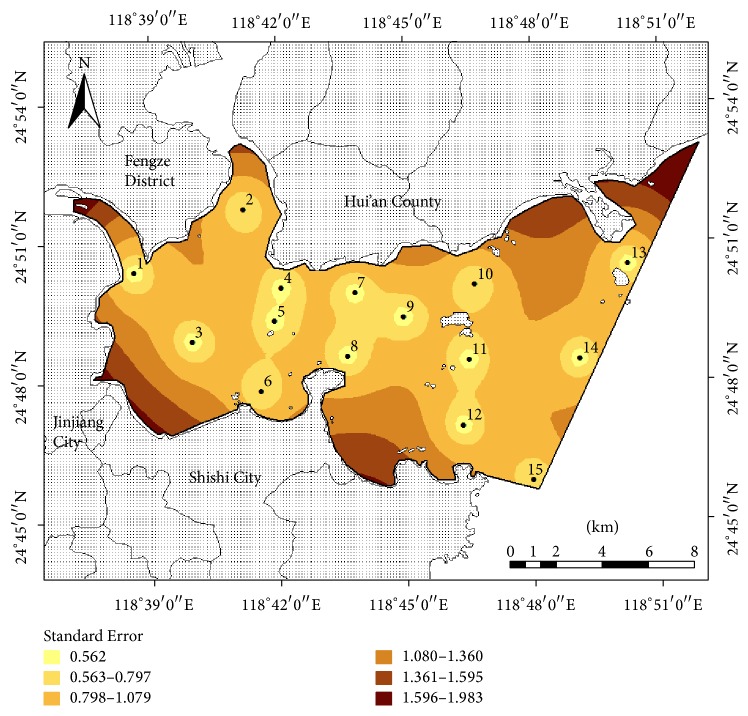
Standard deviation map of the existing coastal environmental monitoring network of Quanzhou Bay.

Based on the eutrophication status of Quanzhou Bay, the values of the Trophic State Index were used to predict the standard deviation of estimated errors at any point using the Kriging method. The average standard deviation of the existing coastal environmental monitoring network of Quanzhou Bay was 1.0231 (Figure 3).

The standard deviations in the open water of Quanzhou Bay were lower than those near the shore; those in the outer bay were lower than those in the inner bay; and those which had dense monitoring sites were smaller than the sparse areas. The reasons for these phenomena were as follows: (1) there were intensive human activities and insufficient sewage infrastructure around Quanzhou Bay, which resulted in the land-based nonpoint source pollutants being directly discharged from the south and west coasts. The sampling sites in the existing network could not cover key areas of pollutant discharge, especially in the southwestern and northeastern Quanzhou Bay, which caused a relatively high standard deviation; and (2) there were relatively dense sampling sites in the inner bay area and the intersection of the Jinjiang River estuary and Luoyang River estuary, which resulted in a relatively small standard deviation.

Based on the existing 15 monitoring sites in Quanzhou Bay, the relationship among the number of monitoring sites, the average standard deviation of the estimated error, and the monitoring precision (compared with 15 sites and *n* − 1 sites) are shown in Table 4. For coastal environmental monitoring network optimization, we need to monitor the coastal environment with a smaller average standard deviation of the monitoring network. For the average standard deviation of the estimated error to decrease from 1.0231 to 0.7143, we need to add an extra 10 sites; and when the average standard deviation of the estimated error is intended to further decrease to below 0.6, this needs the addition of more than 27 sites. The results indicated that based on the special semivariogram model to estimate monitoring network, the average standard deviation of the estimated error is also relevant to the location of the monitoring sites [40]. Under certain budget monitoring, pollution source position, and hydrodynamic conditions, the average standard deviation of the estimated error relates positively to the number and the location of the monitoring sites, and the average standard deviation of the estimated error relates negatively to the density of the monitoring sites.

**Figure 4:**
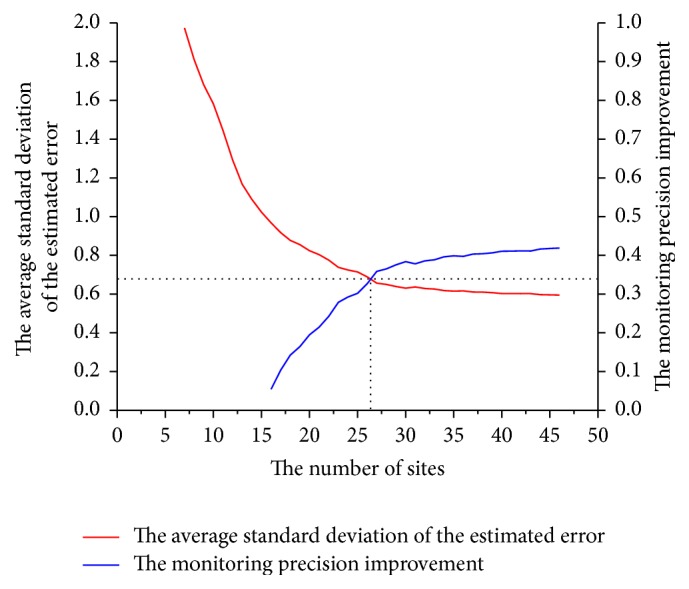
The relationship between the number of sites, the average standard deviation of the estimated error, and monitoring precision improvement (compared with 15 sites).

**Figure 5:**
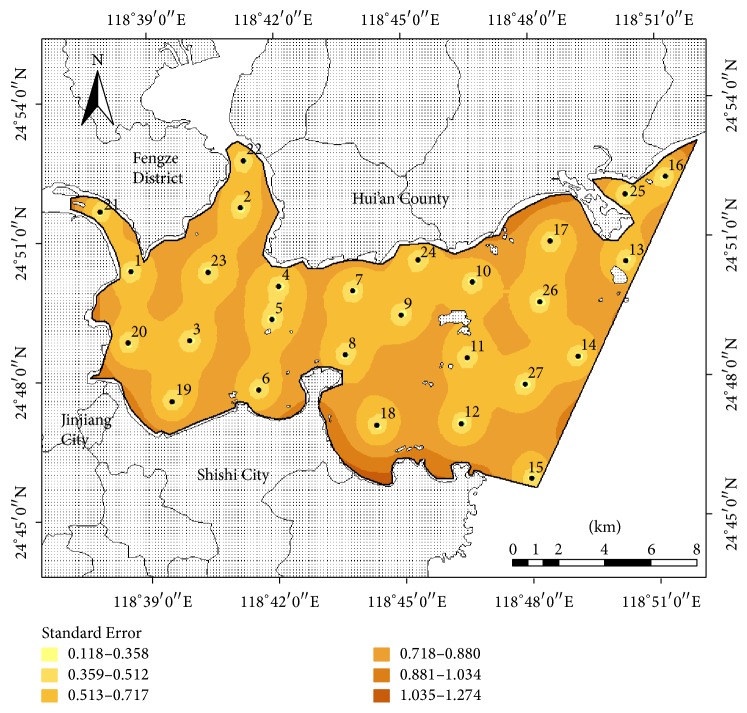
Diagram of the preliminary monitoring network optimization in Quanzhou Bay.

The existing coastal environmental monitoring network had 15 sites, and its average standard deviation of the estimated error was 1.0231. As discussed previously, determining the number of monitoring sites is related to the average standard deviation of the estimated error. However, the increase in number of monitoring sites is limited by the monitoring budget, and the optimal number of sites must be determined by obtaining sufficient environmental information (i.e., meet a certain precision) with acceptable cost.  Figure 4 shows that the intersection point between the curve of the average standard deviation of the estimated error and the curve of the monitoring precision is located between 25 and 30 monitoring sites. Based on Table 4, when the number of sites exceeded 27, the rate of the monitoring precision (compared with *n* − 1 sites) was no longer improved significantly even though more sites were added. Therefore, 27 monitoring sites were determined to be rearranged for the preliminary optimization of the monitoring network as shown in Figure 5. The average standard deviation of the estimated error of the preliminary network optimization was 0.6563, and the monitoring precision increased by 35.85%.

### 3.3. Advanced Optimization of the Monitoring Network in Quanzhou Bay

Although the precision of the monitoring network in Quanzhou Bay has been improved using the Kriging method, the monitoring sites were evenly distributed in the study area without considering the impacts of the hydrodynamic conditions, the outfall setting, and the pollution level. So a prior knowledge and experience would be very important in monitoring network optimization.

The environmental monitoring information would be redundant and unreliable with this preliminary optimization, and so the monitoring network needs to be further improved based on the results. According to the monitoring site design principles of  “the specification for oceanographic survey” and “the specifications for marine monitoring” [57, 58], the monitoring sites for the estuarine area should be designed as fan-shaped distribution along the flow direction in the tidal area; the monitoring sites for the coast area should be designed as denser in the offshore and “key” areas (e.g., sewage outlets, fishery farms, scenic spots, and port terminals) but sparser in the open and “control” areas; and channels, anchorages, dumping zones, and pollution mixing zones should be avoided in the design of the monitoring site. However, the Kriging method will result in the design of excessive monitoring sites in open areas due to the estimated errors at the study area edge [23, 59]. In fact, the hydrodynamic condition and the capacity of seawater self-purification in open areas are better than those in offshore areas. The redundant and unnecessary site designing not only increases the monitoring cost but also provides little more valuable environmental information.

**Figure 6:**
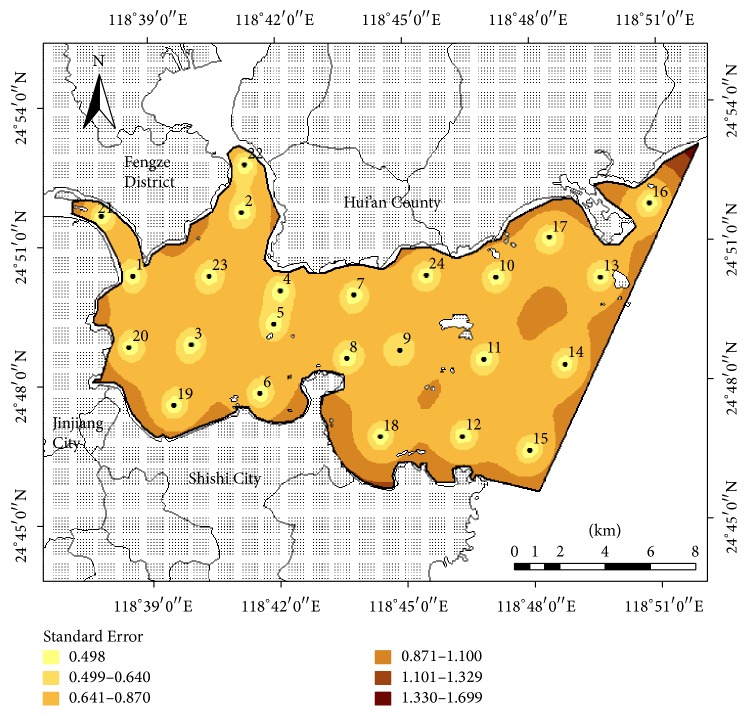
Diagram of the advanced monitoring network optimization in Quanzhou Bay.

The positions of Sites 9–18 and 24 were adjusted to comply with the design principles of the specifications mentioned above. Sites 25–27 were removed because the variation of the environment pollution level in the open area was not obvious based on experience. In consideration of the monitoring sites being denser in the higher eutrophication sea area [57, 60], Sites 19–22 were preserved for their importance on land-based pollutant monitoring although the precision did not improve significantly. The final advanced monitoring network of Quanzhou Bay is shown in Figure 6, and the average standard deviation of the estimated error is 0.6826, with nine new sites having been added to the existing 15 sites. The monitoring precision increased by 32.9%, which was lower than the result of the preliminary optimization. In fact, many monitoring network designs are available, but because of practical limits, the most efficient in theory may not be feasible [24]. The object of the coastal environmental monitoring network optimization was to achieve the environmental information which should meet a certain precision with acceptable cost. As shown in Table 4, to reduce the average standard deviation of the estimated error and improve the monitoring precision, more sites were needed, which meant a monitoring cost increase. In our study, the monitoring network was reliable and the number of sites was trimmed using the advanced optimization, which considered the coastal pollution level and relevant monitoring specifications. Therefore, the final coastal environmental monitoring network of Quanzhou Bay can be considered as satisfying the overall goals.

### 3.4. Comparison with Other Researches in Coastal Areas

In the application of the Kriging method to optimize a coastal environmental monitoring network, the effect is related to reduction of the average standard deviation of the estimated error. In our research, the average standard deviation of the estimated error was lower, the monitoring precision was higher, and the optimized effect was better. Compared with other coastal environmental monitoring network optimization results using the Kriging method (Table 5), the average standard deviation of the estimated error in Quanzhou Bay decreased more than those in the Yangtze River Estuary [23] or Jiaozhou Bay [40].

In the study of the Yangtze River Estuary, Shen and Wu [23] use the Seawater Environmental Quality Index (SEQI) to evaluate the average standard deviation, and the values of SEQI range from 3.646 to 4.809 with a smaller relative interval compared to the Trophic State Index ranging from 0.265 to 7.602 in Quanzhou Bay. Therefore, with the monitoring site density increases, the average standard deviation of the estimated error was reduced less. However, since the SEQI value in one site is equivalent to its highest pollution index and is not obtained from one specific element or a linear combination of the elements, it is difficult to guarantee its spatial continuity and correlation in such a large area [9]. The same result appears in the coastal environmental monitoring network optimization in Jiaozhou Bay [40], where the values of the monitoring elements are standardized and normalized in the range 0.613 to 1.300, which leads to the optimized average standard deviation reducing less.

In another study in the Yangtze River Estuary and its adjacent area conducted by Gao et al. [9], 12 seawater parameters are divided into three groups using principal component analysis to reduce the dimensionality of the environmental variables, and then the mean of surface with nonhomogeneity (MSN) method is used to optimize the marine environmental monitoring network. The MSN method performs well in the Yangtze River Estuary and its adjacent area, owing to the apparent spatially stratified heterogeneity and spatial autocorrelation [27]. However, it is not easy nor suitable to apply it in a semienclosed bay without the complicated hydrological elements.

Cao et al. [41] find that some of the existing coastal environmental monitoring sites in Xiangshan Bay are redundant, based on the same monitoring precision to reduce and to adjust sites in order to achieve network optimization and cost cutting. However, whether the optimized network fully reflects the environmental information in the highly polluted area remains to be tested.

## 4. Conclusions

An efficient monitoring network is very important for coastal environmental quality assessment, protection, and management. Reliable water quality information analysis would help to further optimize the coastal environment monitoring network and benefit data analysis, risk assessment, and reporting [11]. In our study, we used the Trophic State Index to assess the coastal environmental pollution level in Quanzhou Bay, a semienclosed bay. We concluded that Quanzhou Bay had a high level of nutrient pollution and that the inner bay area suffered more serious eutrophication issues than the open area. Based on the results of the Trophic State Index, we used Geographical Information System software and the Kriging interpolation method to evaluate the quality of the existing coastal environmental monitoring network and to optimize the design of the monitoring network in Quanzhou Bay. Considering the average standard deviation of the estimated error, the coastal pollution level, and the hydrodynamic conditions of Quanzhou Bay, the number of monitoring sites was increased from 15 to 24 with nine new ones added and six old ones slightly adjusted, thus improving the monitoring precision by 32.9%. Our study suggested an optimal design of the monitoring network in Quanzhou Bay, and the method is very practical, user-friendly, and cost-effective in this sea area.

This stage position cannot be accomplished in one optimization, however, and the looping process requires more work to adjust and verify. For example, if the size of spatial data is large, the phenomenon of spatial stratified heterogeneity should be considered, which implies the existence of distinct mechanisms by strata may affect the performance by Kriging method [61]. Other suggestions should be valuable in monitoring network design: adjusting monitoring site positions and improving the monitoring precision based on historical data; optimizing the monitoring network within specific time and space; enhancing the support for coastal management departments; and consulting the monitoring network design in other aquatic systems.

## Figures and Tables

**Table 1:** Mean values of COD, DIN, and PO_4_-P and the Trophic State Index in the seawater of Quanzhou Bay from 2009 to 2012 (mg/L).

Site	COD	DIN	PO_4_-P	Trophic State Index
1	1.314	2.186	0.071	7.602
2	1.184	1.929	0.047	3.947
3	1.141	1.617	0.049	3.381
4	0.924	1.319	0.040	1.799
5	1.056	1.661	0.049	3.209
6	1.158	1.674	0.062	4.444
7	0.776	1.171	0.033	1.124
8	0.807	1.332	0.043	1.694
9	0.591	0.896	0.032	0.621
10	0.566	0.716	0.032	0.477
11	0.543	0.736	0.027	0.402
12	0.513	0.896	0.031	0.520
13	0.574	0.744	0.023	0.369
14	0.556	0.762	0.024	0.376
15	0.483	0.662	0.022	0.265

**Table 2:** Seawater quality standards and categories in China^*∗*^.

Quality grade		I	II	III	IV
Seawater quality index	COD (mg/L)	0–2	2-3	3-4	4-5
	DIN (mg/L)	0–0.20	0.20–0.30	0.30–0.40	0.40–0.50
	PO_4_-P (mg/L)	0–0.15	0.015–0.030	0.015–0.030	0.030–0.045

^*∗*^Seawater quality standard from state oceanic administration [39].

**Table 3:** The prediction errors of the semivariogram modeling.

Model	Mean Standardized	Root-Mean-Square	Average Standard Error	Root-Mean-Square Standardized
Circular	0.0175	0.7497	0.0061	0.4476
Spherical	0.0075	0.6919	0.0012	0.5917
Tetraspherical	0.0223	0.7451	0.0158	0.5074
Exponential	0.0330	0.7131	0.0840	0.4889

**Table 4:** The influence of the number of monitoring sites on the average standard deviation of the estimated error and the rate of the monitoring precision.

The number of the sites	The average standard deviation of estimated error	The rate of the monitoring precision (compared with 15 sites)	The rate of the monitoring precision (compared with *n* − 1 sites)	The number of the sites	The average standard deviation of estimated error	The rate of the monitoring precision (compared with 15 sites)	The rate of the monitoring precision (compared with *n* − 1 sites)
15	1.0231			31	0.6364	37.80%	−0.95%
16	0.9675	5.43%	5.43%	32	0.6285	38.57%	1.24%
17	0.9175	10.32%	5.17%	33	0.6257	38.84%	0.45%
18	0.8774	14.24%	4.37%	34	0.6179	39.61%	1.25%
19	0.8549	16.44%	2.56%	35	0.6151	39.88%	0.45%
20	0.8238	19.48%	3.64%	36	0.6165	39.74%	−0.23%
21	0.8037	21.44%	2.44%	37	0.6107	40.31%	0.94%
22	0.7751	24.24%	3.56%	38	0.6098	40.40%	0.15%
23	0.7379	27.88%	4.80%	39	0.6074	40.63%	0.39%
24	0.7242	29.22%	1.86%	40	0.6029	41.07%	0.74%
25	0.7143	30.18%	1.37%	41	0.6026	41.10%	0.05%
26	0.6889	32.67%	3.56%	42	0.6023	41.13%	0.05%
27	0.6563	35.85%	4.73%	43	0.6024	41.12%	−0.02%
28	0.6496	36.51%	1.02%	44	0.5972	41.63%	0.86%
29	0.6388	37.56%	1.66%	45	0.5958	41.77%	0.23%
30	0.6304	38.38%	1.31%	46	0.5947	41.87%	0.18%

**Table 5:** Comparison of the coastal environmental monitoring network optimization with other studies.

Study area	The number of original sites	The number of optimized sites	The number of added sites	The number of deleted sites	The rate of the monitoring precision	Source
Yangtze River Estuary	42	59	21	4	16.1%	Shen and Wu, 2013 [23]
Yangtze River Estuary	70	55	5	20	It depends	Gao et al., 2015 [9]
Jiaozhou Bay	28	31	6	3	8.3%	Yi et al., 2014 [40]
Xiangshan Bay	50	38	0	12	0	Cao et al., 2014 [41]
Quanzhou Bay	15	24	12	3	32.9%	This study
